# Neural mechanism of HT7 electroacupuncture in myocardial ischemia: critical role of the paraventricular nucleus oxytocin system

**DOI:** 10.3389/fnins.2025.1678938

**Published:** 2025-10-09

**Authors:** Hao-Sheng Wu, Li Zhu, Pan Liu, Xiao-Hui Liu, Hang Su, Xin-Yi Zheng, Tong-Tong Liu, Shuai Cui, Sheng-Bin Wu, Mei-Qi Zhou, Chao Zhu

**Affiliations:** ^1^College of Chinese Medicine, Anhui University of Chinese Medicine, Hefei, China; ^2^College of Acupuncture and Massage, Anhui University of Chinese Medicine, Hefei, China; ^3^Anhui Province Key Laboratory of Meridian Viscera Correlationship, Hefei, China

**Keywords:** myocardial ischemia, electroacupuncture, Shenmen (HT7), paraventricular nucleus, oxytocinergic neurons

## Abstract

**Objective:**

Electroacupuncture (EA) at Shenmen (HT7) alleviates acute myocardial ischemia (AMI), but its central neuromodulatory mechanisms remain insufficiently defined. This study investigated whether oxytocinergic (OT) neurons in the hypothalamic paraventricular nucleus (PVN) mediate the cardioprotective effects of EA and verified causality through chemogenetic interventions.

**Methods:**

Part I: C57BL/6 mice (*n* = 18) were allocated into three groups: sham, AMI, and AMI + EA-HT7 (2 Hz/1 mA, 30 min/day × 3 days). Cardiac function (echocardiography), histopathology (HE/Masson staining), and PVN electrophysiology were evaluated. Part II: C57BL/6 mice (*n* = 18) were divided into sham, AMI, and AMI + EA-HT7 groups. Mice received AAV-hSyn-DIO-mCherry and AAV-cre-OT injections into the PVN. EA-induced neuronal activation was quantified by c-Fos and OT co-localization. Part III: C57BL/6 mice (*n* = 18) were assigned to chemogenetic activation (OT-activation), inhibition (OT-inhibition), and control (OT-control) groups. Under physiological conditions, the effects of chemogenetic modulation on PVN electrophysiology, electrocardiography, and echocardiography were assessed. Part IV: C57BL/6 mice (*n* = 24) were divided into AMI + OT-control, AMI + EA-HT7 + OT-control, AMI + OT-inhibition, and AMI + EA-HT7 + OT-activation groups. Under pathological conditions, the effects of chemogenetic regulation on PVN physiology and cardiac function were examined.

**Results:**

EA at HT7 improved left ventricular ejection fraction (*p* < 0.05), correlating with suppression of PVN neuronal firing. EA specifically inhibited PVN^OT^ neuronal activity (*p* < 0.05). Under physiological conditions, chemogenetic modulation of PVN^OT^ neurons altered local electrophysiological activity and cardiac performance. In AMI mice, OT neuronal activation reversed EA-induced cardioprotection (*p* < 0.05).

**Conclusion:**

PVN oxytocinergic neurons are both necessary and sufficient for the cardioprotective effects of EA at HT7. Chemogenetic regulation confirms a causal neurocardiac axis, providing mechanistic insights that support precision acupuncture therapies for ischemic heart disease.

## Introduction

Myocardial ischemia (MI), a critical manifestation of coronary artery disease, remains a leading cause of morbidity and mortality worldwide despite advances in pharmacological and interventional therapies (Gibbons, 2021). The pathophysiological cascade—including oxidative stress, mitochondrial dysfunction, and inflammatory activation—contributes to irreversible cardiomyocyte apoptosis, underscoring the urgent need for novel cardioprotective strategies (Gumpper-Fedus et al., 2022; Ramachandra et al., 2020; Algoet et al., 2023).

Recent evidence indicates that oxytocinergic neurons in the hypothalamic PVN are significantly activated in models of myocardial infarction, suggesting a potential role in disease pathogenesis; however, the underlying mechanisms remain unclear (Roy et al., 2018). Moreover, studies have demonstrated that hypothalamic oxytocin (OT) levels rise in multiple cardiovascular nuclei and peripheral tissues in response to various stressors, including hypoxia (Dantzler and Kline, 2020).

Electroacupuncture, a modality rooted in traditional Chinese medicine and increasingly integrated into contemporary clinical practice, shows promising cardiovascular effects (Ma et al., 2014). Shenmen (HT7), a well-established acupoint on the heart meridian, has been widely applied in the treatment of cardiac disorders (Kun et al., 2023). Preclinical studies suggest that EA at HT7 modulates autonomic function and reduces ischemic arrhythmias (Huang et al., 2015); however, the precise neurobiological mechanisms, particularly the involvement of PVN^OT^ signaling, remain largely unexplored.

This study tested the hypothesis that EA at HT7 attenuates myocardial ischemic injury by modulating oxytocinergic neurons in the PVN, thereby engaging cardioprotective pathways. By integrating chemogenetics, viral neuronal tracing, and molecular profiling, we quantified EA-induced activation of PVN^OT^ neurons during AMI and assessed their necessity and sufficiency in mediating cardioprotection. The findings establish a novel HT7-PVN^OT^-heart axis, providing mechanistic evidence for acupuncture as an adjunctive therapy in ischemic heart disease.

## Materials and methods

### Animals

C57BL/6 J mice were obtained from Hangzhou Ziyuan Experimental Animals Technology Co., Ltd. (Certificate No. SCXK-Zhe-2024-0004). All animals were housed under specific pathogen-free (SPF) conditions at a controlled temperature (22 ± 1 °C) with a 12 h light/dark cycle. Food and water were provided ad libitum. After 1 week of acclimatization, experimental procedures were initiated.

Four independent cohorts were established: Cohort I (*n* = 18): Sham (*n* = 6), AMI (*n* = 6), AMI + EA-HT7 (*n* = 6). Cohort II (*n* = 18): Sham (*n* = 6), AMI (*n* = 6), AMI + EA-HT7 (*n* = 6). Cohort III (*n* = 18): OT-activation (*n* = 6), OT-inhibition (*n* = 6), OT-control (*n* = 6).

Cohort IV (*n* = 24): AMI + OT-control (*n* = 6), AMI + EA-HT7 + OT-control (*n* = 6), AMI + OT-inhibition (*n* = 6), AMI + EA-HT7 + OT-activation (*n* = 6).

All procedures were approved by the Animal Ethics Committee of Anhui University of Traditional Chinese Medicine (Approval No. AHUCM-mouse-2024146).

### Acute myocardial infarction model

**Figure 1:**
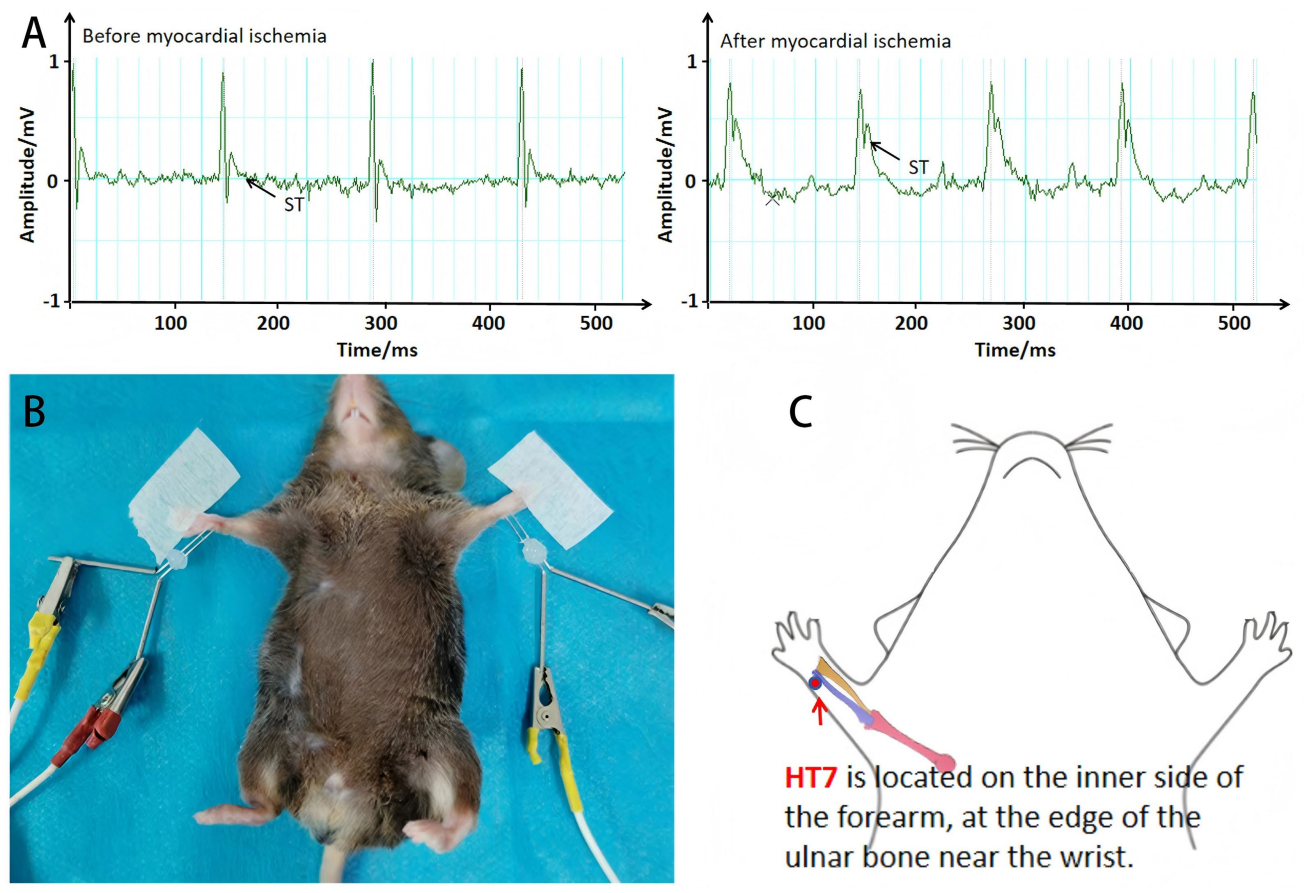
Schematic diagram of AMI modeling and EA intervention. **(A)** Electrocardiographic ST-segment changes before and after LAD ligation. **(B)** Photograph of mice undergoing EA at HT7 (2 Hz, 1 mA, depth 1 mm, vertical insertion). **(C)** Anatomical localization of HT7.

Mice were anesthetized in an induction chamber with 3% isoflurane and subsequently maintained under 1–1.5% isoflurane. After shaving and disinfecting the thoracic region, a left thoracotomy was performed at the fourth intercostal space to expose the heart. The left anterior descending (LAD) coronary artery was ligated with a 6–0 suture at the site where blanching of the myocardium was observed. After ligation, the thoracic cavity was closed, residual air expelled, and penicillin administered to prevent infection. Successful AMI was confirmed by ST-segment elevation >0.1 mV on electrocardiography (Peng et al., 2024) (Figure 1A). Sham controls underwent threading without ligation.

### Electroacupuncture intervention

EA was administered immediately after AMI induction in a quiet environment. Acupuncture needles were vertically inserted (depth: 1 mm) into bilateral Shenmen (HT7), located at the ulnar side of the wrist crease (China Association of Acupuncture-Moxibustion, 2021). A continuous wave stimulus (2 Hz, 1 mA) was delivered for 30 min/day over 3 consecutive days (Figures 1B,C). Sham and AMI groups did not receive EA.

### Animal anesthesia and euthanasia

All experiments adhered to the principle of minimizing animal pain and distress. During model establishment, electroacupuncture intervention, electrophysiological testing, and stereotactic brain injections, gas anesthesia with isoflurane was administered. Animals were first placed in an induction chamber and anesthetized with 3% isoflurane. Once immobile, they were transferred to an anesthesia mask, and the concentration was adjusted to 1–1.5% to maintain anesthesia. Euthanasia was performed using 5% isoflurane inhalation for more than 1 min. The absence of heartbeat and respiration confirmed death. The carcasses were transported to the designated recycling facility of the Laboratory Animal Center, Anhui University of Traditional Chinese Medicine.

### HE staining

Hematoxylin–eosin (HE) staining was used to evaluate pathological changes in myocardial tissue. Samples were fixed in 4% paraformaldehyde for 24 h at 4 °C, followed by dehydration, embedding, sectioning, dewaxing, and hematoxylin and eosin staining. Slides were sealed and examined under a bright-field microscope for morphological assessment.

### Masson staining

After collection, tissues were fixed in 4% neutral formaldehyde for 24–48 h and embedded in paraffin to prepare 4–5 μm sections, which were baked at 60 °C for 2 h. Sections were deparaffinized in xylene I and II (10 min each), rehydrated in absolute ethanol (5 min), and sequentially immersed in graded ethanol (95, 80, 70%; 2 min each), followed by rinsing in distilled water. Nuclear staining was performed with Weigert’s iron hematoxylin (5–10 min), rinsed (10 min), differentiated in 1% HCl ethanol, and re-blued with lithium carbonate. Cytoplasmic staining was performed with lilac acid fuchsin (5 min), followed by 1% phosphomolybdic acid differentiation (3 min). Collagen fibers were stained with aniline blue or light green (5 min), briefly rinsed in 1% acetic acid, dehydrated in 95% ethanol and absolute ethanol I/II (2 min each), cleared in xylene (5 min), and mounted with neutral resin.

### Immunofluorescent staining

Frozen sections were washed three times with PBS (5 min each) and permeabilized with 0.5% Triton X-100 for 30 min at room temperature. Sections were blocked with 5% goat serum for 2 h at room temperature. Primary antibodies diluted in blocking buffer (1:500) were applied to cover the tissue, and slides were incubated overnight at 4 °C in a humidified, light-protected chamber. After three washes (10 min each), fluorescently labeled secondary antibodies (1:500) were applied for 2 h at room temperature in the dark. Slides were washed, counterstained with DAPI for 5–10 min, mounted with an anti-fade medium, and sealed with nail polish. Imaging was performed using the PanoBrain slide scanner.

### Electrocardiogram

Electrocardiography was recorded using the PowerLab system to monitor cardiac function. Electrodes were implanted subcutaneously in the right forelimb and left hindlimb, and intramuscularly in the right hindlimb. Once stable waveforms were obtained, recordings were continued to assess heart rate (HR) and heart rate variability (LF/HF ratio).

### Echocardiography

Left ventricular function was assessed by echocardiography. Parameters measured included ejection fraction (EF, %), fractional shortening (FS, %), left ventricular end-diastolic diameter (LVIDd, mm), left ventricular end-systolic diameter (LVIDs, mm), left ventricular end-diastolic volume (LVEDV, mL), and left ventricular end-systolic volume (LVESV, mL).

### Electrophysiology

After anesthesia, mice were placed prone, and their heads were secured in a stereotaxic apparatus. The scalp was shaved and disinfected with iodophor, and a midline incision was made. Connective tissue was separated to expose the skull surface. Anterior and posterior fontanelles were identified using sutural landmarks, and the head was adjusted so that deviations were within 0.02 mm. A cranial hole (0.3 mm diameter) was drilled above the PVN target site. A microelectrode was advanced into the PVN, and the electrode was fixed in place with dental cement.

### Micro-injection

Following anesthesia with isoflurane (induction 3%, maintenance 1%), mice were fixed in a stereotaxic instrument. Ear bars were positioned symmetrically, ensuring horizontal alignment of the skull. Proper fixation was confirmed by alignment of the nose, absence of head movement, and horizontal placement of the skull. After shaving and disinfecting the scalp, a midline incision was made, and the dura was removed to expose Bregma. Coordinates relative to Bregma were: AP –1.07 mm, ML ± 0.24 mm, DV –4.8 mm. A small burr hole was drilled above the PVN, and 20 nL of viral vector was injected using an RWD microinjector at 0.15 μL/min. The needle was left in place for 5–10 min to prevent reflux.

### Statistical analysis

Data analysis and graphs were generated with GraphPad Prism 8.0. Data are presented as mean ±standard deviation. The normality test adopts the Shapiro–Wilk test. The homogeneity of variance employs the Brown-Forsythe test. The data for comparison between the two groups were first subjected to normality tests. Those that met the normality criteria were analyzed using the Student’s *T*-test or Paired *T*-test. The data that do not conform to normality undergo the Mann–Whitney test or Wilcoxon test. Perform normality and homogeneity of variance tests on the experimental data from multiple groups for comparison. If both normality and homogeneity of variance are satisfied, a one-way analysis of variance should be used for the test; if not, the Kruskal-Wallis H rank sum test should be conducted, followed by Dunn’s multiple comparison for the analysis between groups. Correlations were evaluated using Pearson’s correlation coefficient. A value of *p* < 0.05 was considered statistically significant.

## Results

### Effect of electroacupuncture at HT7 on myocardial injury and cardiac function

**Figure 2:**
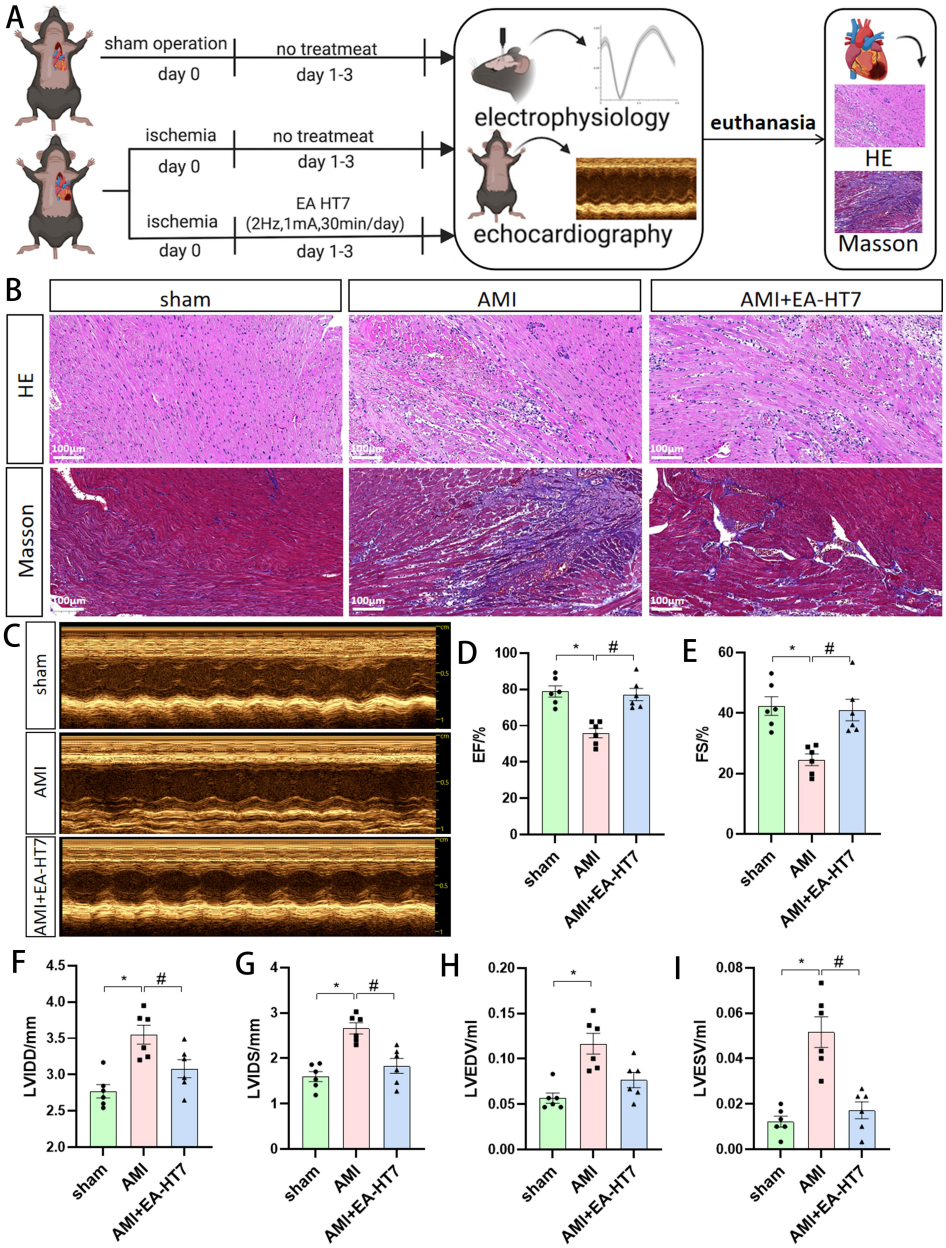
Effect of electroacupuncture at HT7 on myocardial injury and cardiac function. **(A)** Flowchart of the effect of electroacupuncture on improving cardiac function in mice with myocardial ischemia. After the model was established, the intervention was applied for three consecutive days. Electrophysiological and echocardiographic examinations were performed about 30 min after the final intervention. Following these *in vivo* assessments, euthanasia was conducted, and the hearts were collected for hematoxylin–eosin (HE) and Masson staining. **(B)** Representative images of HE and Masson staining. **(C)** Echocardiographic image. **(D–I)** Effects of electroacupuncture on indicators of cardiac function in mice with myocardial ischemia. Data are expressed as mean ±standard deviation (biological replicates *n* = 6 rats/group). Compared with the sham group, * *p* < 0.05; compared with the AMI group, # *p* < 0.05.

By examining the effects of different interventions on myocardial injury and cardiac function (Figure 2A), it was observed that compared with the sham group, the AMI group showed marked myocardial structural damage, disordered fibers, and inflammatory cell infiltration in HE staining. Masson staining revealed increased collagen fiber deposition (blue), indicating myocardial injury and fibrosis in AMI (Figure 2B). Echocardiography revealed a significant decrease (*p* < 0.05) in ejection fraction (EF) and shortening fraction (FS), along with significant increases (*p* < 0.05) in left ventricular end-diastolic diameter (LVIDD), left ventricular end-systolic diameter (LVIDS), left ventricular end-diastolic volume (LVEDV), and left ventricular end-systolic volume (LVESV). Compared with the AMI group, HT7 electroacupuncture treatment led to recovery of myocardial tissue structure, reduced inflammatory cell infiltration, alleviated collagen deposition, significantly increased EF and FS (*p* < 0.05), and markedly decreased LVIDD, LVIDS, and LVESV (*p* < 0.05). These findings indicate that stimulation at HT7 alleviated myocardial injury and left ventricular dysfunction induced by AMI (Figures 2C–I).

### Effect of electroacupuncture at HT7 on brain wave activity in the PVN

**Figure 3:**
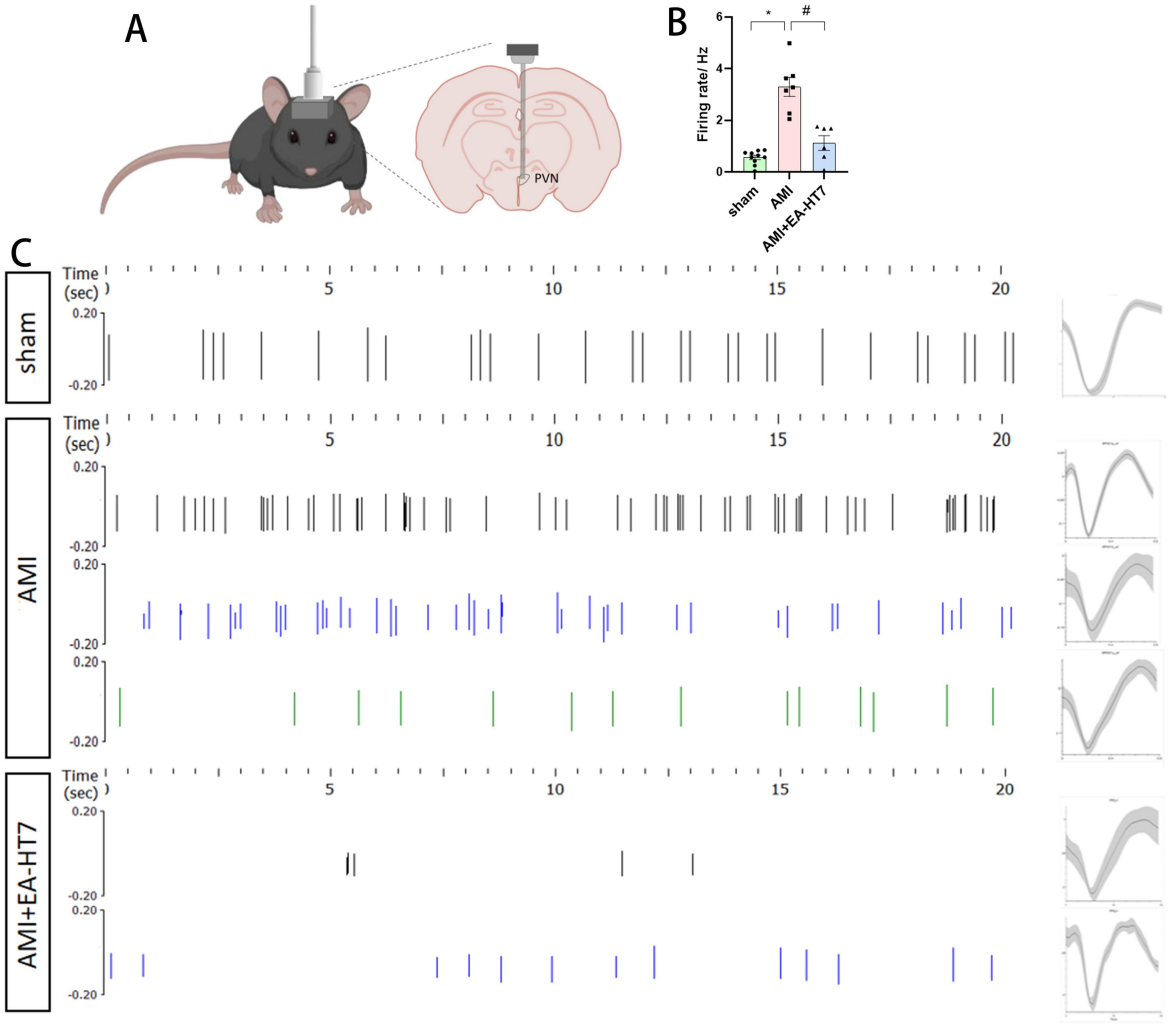
Effect of electroacupuncture at HT7 on brain waves of the PVN. **(A)** Schematic diagram showing PVN implantation with microfilament electrodes. **(B)** Influence of electroacupuncture on neuronal firing frequency. **(C)** Spike discharge raster diagram (left) and neuronal morphology (right). Data are expressed as mean ±standard deviation (biological replicates *n* = 3 rats/group, each group of records contains at least 6 valid signals from the channels). Compared with the sham group, * *p* < 0.05; compared with the AMI group, # *p* < 0.05.

By examining the effects of different interventions on neuronal discharges in the paraventricular nucleus (PVN) of the hypothalamus, it was found that, compared with the sham operation group, the number of spike discharges in the AMI group significantly increased (*p* < 0.05), indicating activation of the PVN during AMI. In contrast, the number of spike discharges significantly decreased after HT7 treatment (*p* < 0.05) (Figure 3), suggesting that electroacupuncture at HT7 inhibited neuronal activity in the hypothalamic PVN.

### Electroacupuncture-mediated cardiac functional improvement is closely associated with cerebral electrical activity

**Figure 4:**
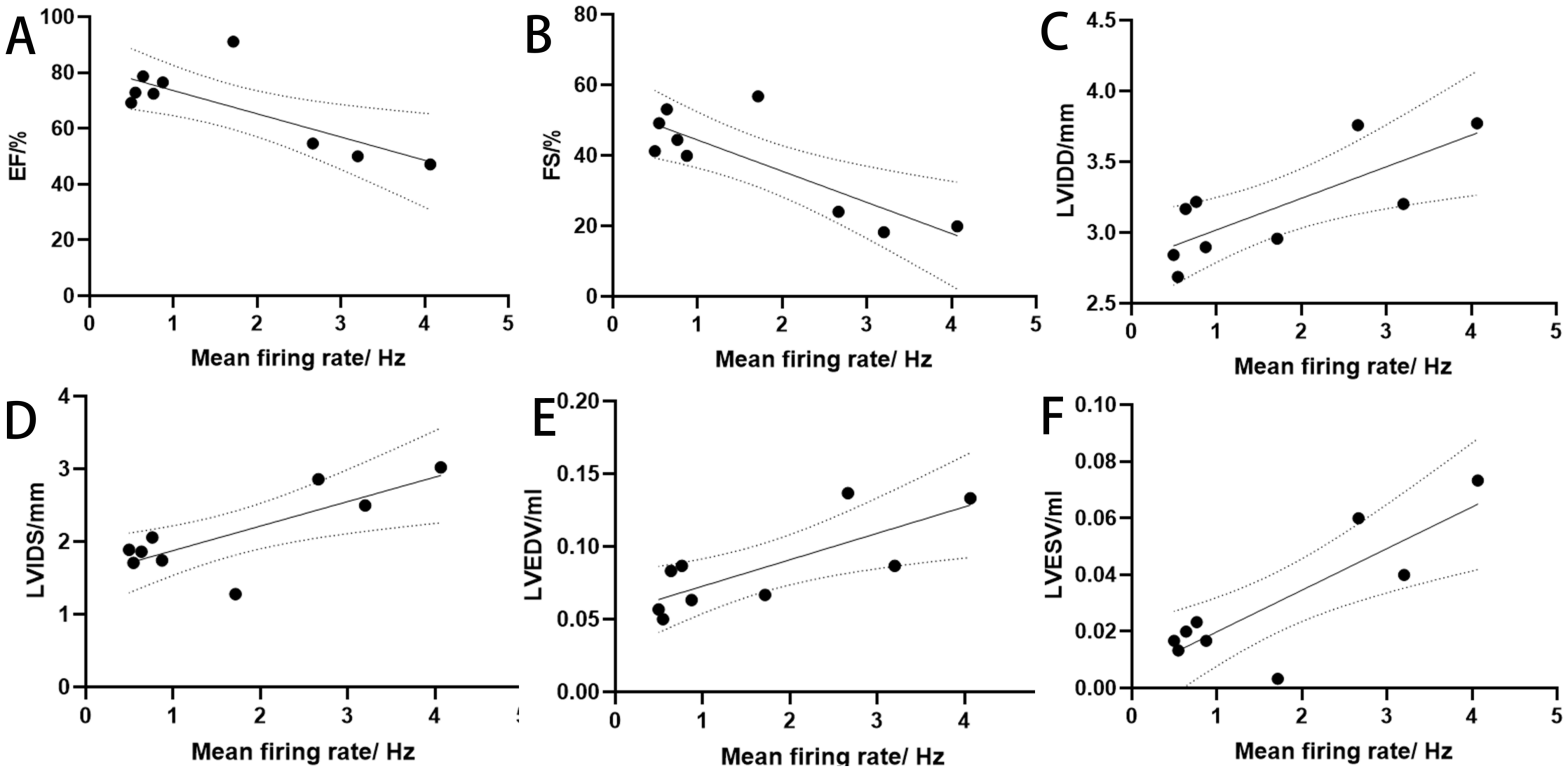
Correlation analysis of cardiac function and discharge frequency. **(A)** Y = −8.321*X + 81.91, R = –0.7609. **(B)** Y = −8.843*X + 53.23, R = –0.8167. **(C)** Y = 0.2231*X + 2.796, R = 0.7770. **(D)** Y = 0.3368*X + 1.541, R = 0.7818. **(E)** Y = 0.01817*X + 0.05460, R = 0.7748. **(F)** Y = 0.01468*X + 0.005225, R = 0.8389 (biological replicates *n* = 3 rats/group).

A correlation analysis was performed between cardiac function indicators and spike discharges of the PVN during HT7 intervention. The results demonstrated that changes in cardiac function were significantly correlated with PVN neuronal discharge (*p* < 0.05) (Figure 4).

### Changes in oxytocinergic neurons in PVN by electroacupuncture HT7

**Figure 5:**
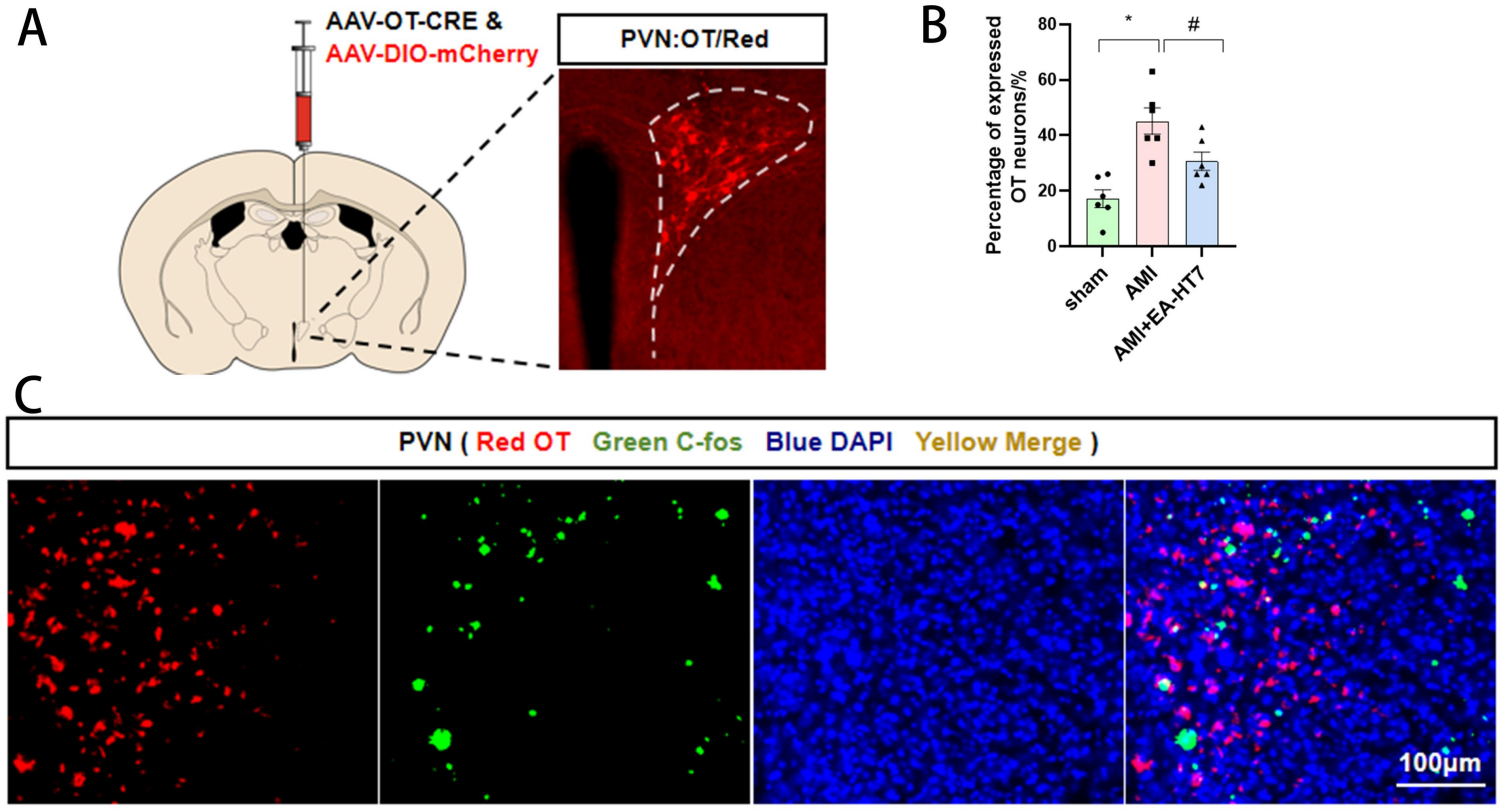
Effect of electroacupuncture HT7 on the oxytocin neurons of PVN. **(A)** Schematic diagram of virus injection and site localization. **(B)** Co-localization of OT neurons and c-fos. **(C)** Percentage of c-fos-positive OT neurons among total OT neurons. Data are expressed as mean ±standard deviation (biological replicates *n* = 6 rats/group). Compared with the sham group, * *p* < 0.05; compared with the AMI group, # *p* < 0.05.

OT neurons were labeled using an adeno-associated virus (AAV), followed by c-fos immunofluorescence detection (Figures 5A,B). OT neurons were found to be co-localized with c-fos (Figure 5B), suggesting that oxytocinergic neurons in the PVN were activated during myocardial ischemia. Furthermore, the expression of activated OT neurons significantly increased in the AMI group (*p* < 0.05). In contrast, after HT7 intervention, the number of activated OT neurons was significantly reduced (*p* < 0.05) (Figure 5C). These results indicate that oxytocinergic neurons in the PVN may participate in the mechanism by which electroacupuncture alleviates myocardial ischemia.

### Changes in the brain wave of PVN by regulating oxytocinergic neurons in PVN

**Figure 6:**
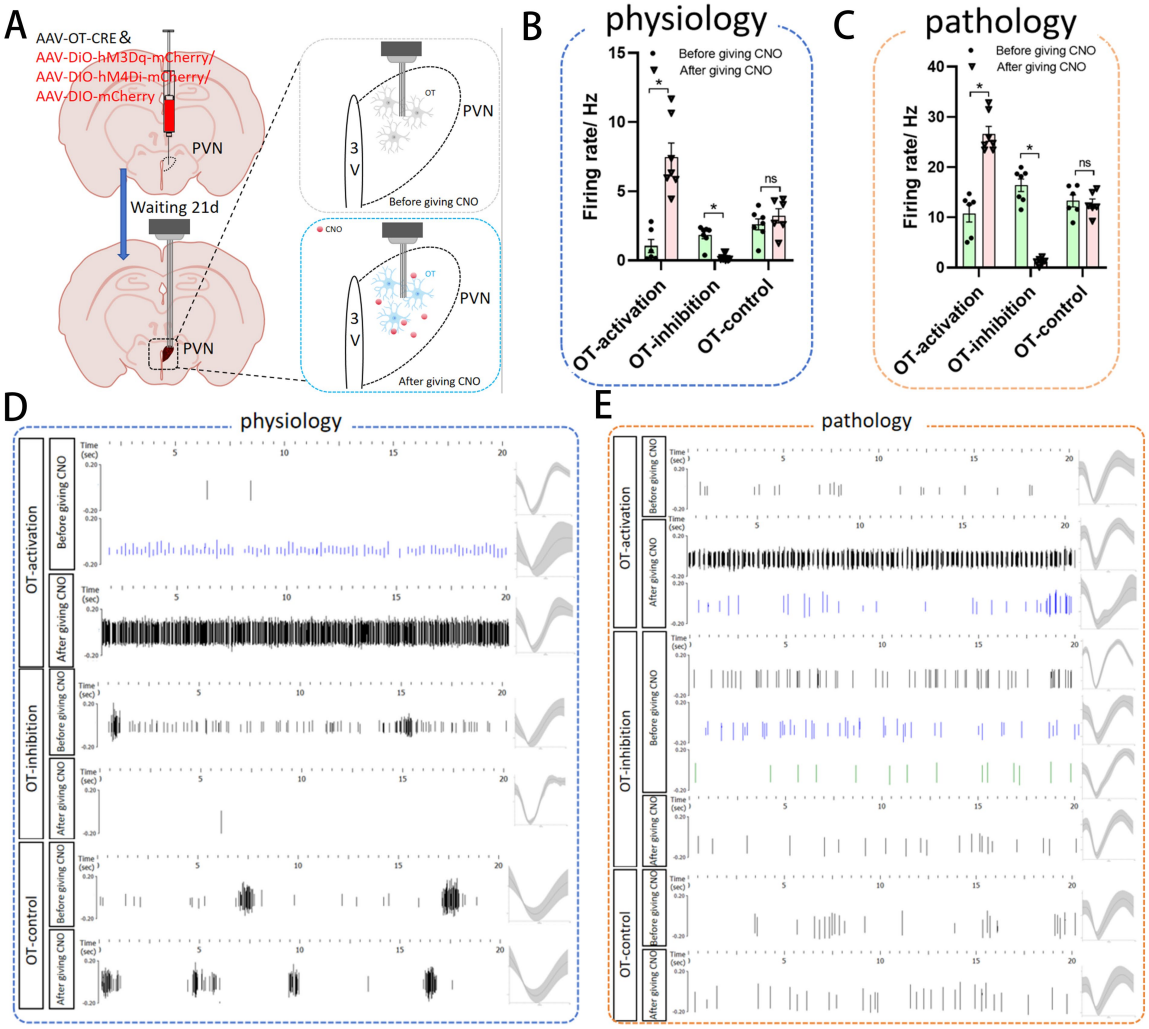
Changes in the brain wave of PVN by regulating oxytocinergic neurons in PVN. **(A)** Flowchart of virus injection and electrophysiological procedure: after injecting the chemogenetic virus into the PVN, the virus was allowed to express for 14 days. Electrophysiological electrodes were then implanted. At 21 days post-injection, clozapine-N-oxide (CNO) was administered intraperitoneally to activate the virus, and neuronal discharges of the PVN were recorded 30 min later. **(B)** Neuronal firing frequency of the PVN under physiological conditions following neuronal regulation. **(C)** Neuronal firing frequency of the PVN under pathological conditions following neuronal regulation. **(D)** Firing frequency and waveform of PVN neurons under physiological conditions. **(E)** Firing patterns and waveform of PVN neurons under pathological conditions. Data are expressed as mean ±standard deviation (biological replicates *n* = 3 rats/group, each group of records contains at least 6 valid signals from the channels). * *p* < 0.05 indicates a significant difference between groups.

Chemogenetic regulation of OT neurons was performed to assess their role in PVN activity (Figure 6A). Under normal physiological conditions, activation of OT neurons significantly increased the neuronal discharge level of the PVN (*p* < 0.05), whereas inhibition of OT neurons significantly reduced PVN neuronal discharge (*p* < 0.05) (Figures 6B,D). In the AMI pathological model, activation of OT neurons also elevated the neuronal discharge level of the PVN (*p* < 0.05), whereas inhibition of OT neurons significantly reduced PVN neuronal discharge (*p* < 0.05) (Figures 6C,E).

### Changes in cardiac function by regulating oxytocinergic neurons in PVN

**Figure 7:**
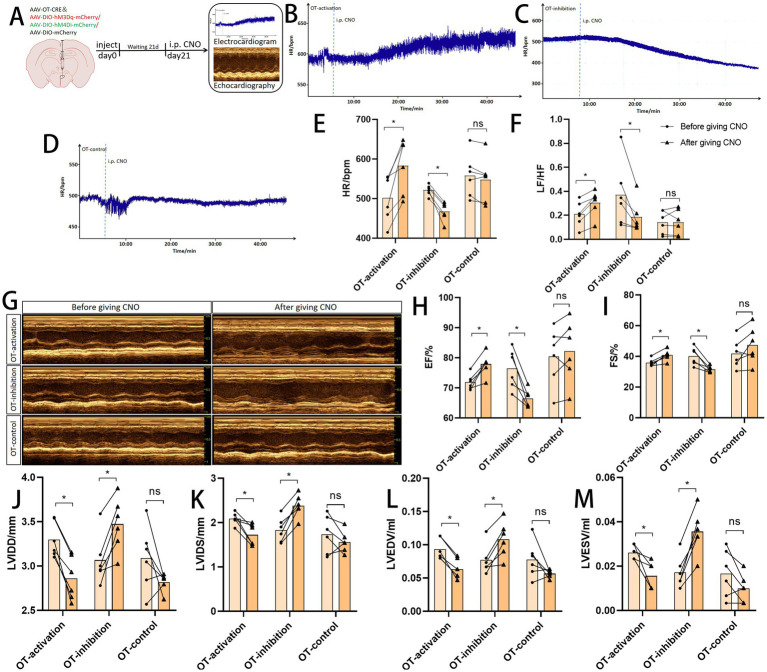
Cardiac function by regulating oxytocinergic neurons in PVN. **(A)** Flowchart of virus injection and cardiac function testing: after injecting the chemogenetic virus into the PVN, the virus was allowed to express for 21 days. Following intraperitoneal (i.p.) injection of clozapine-N-oxide (CNO), a 30 min interval was observed before conducting electrocardiography and echocardiography. **(B–E)** Influence of oxytocinergic neurons in the paraventricular nucleus (PVN) of the hypothalamus on heart rate. **(F)** Heart rate variability (LF/HF ratio). **(G)** Schematic illustration showing regulation of cardiac function by oxytocinergic neurons in the PVN. **(H–M)** Effects of PVN oxytocinergic neuron regulation on cardiac function indicators. Data are presented as mean ±standard deviation (biological replicates *n* = 6 rats/group). * *p* < 0.05 indicates a significant difference between groups.

Under normal physiological conditions, activation of OT neurons significantly increased heart rate (HR) and heart rate variability (*p* < 0.05), whereas inhibition of OT neurons significantly reduced HR and heart rate variability (*p* < 0.05) (Figures 7B–F). Furthermore, activation of OT neurons enhanced left ventricular function (*p* < 0.05), while inhibition of OT neurons reduced left ventricular function (*p* < 0.05) (Figures 7H–M). These findings suggest that OT neurons in the PVN may play a regulatory role in cardiac function (Figures 7A,G).

### Activation of oxytocinergic neurons in PVN can reverse the beneficial effects of electroacupuncture HT7

**Figure 8:**
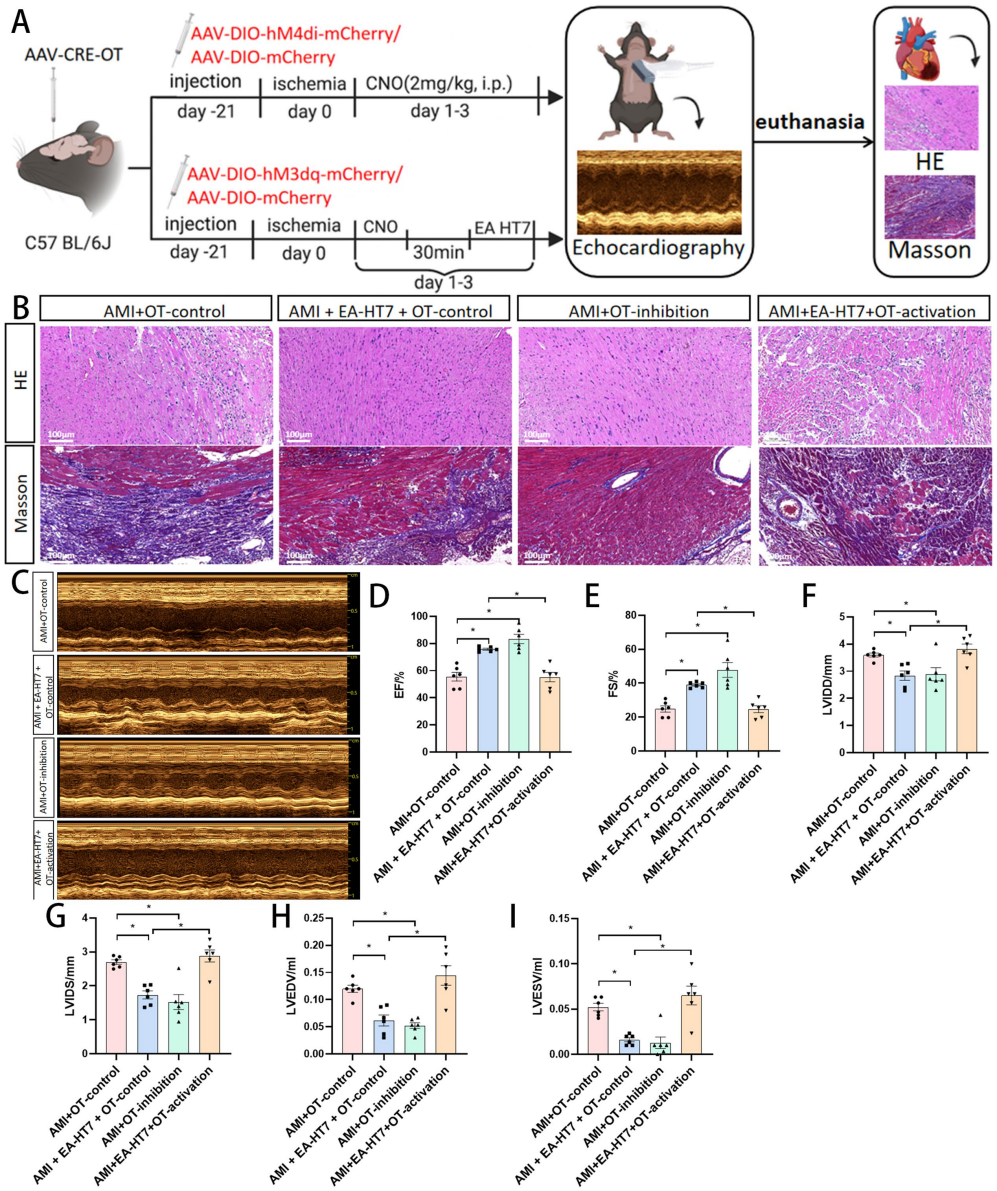
Cardiac function in mice with myocardial ischemia by regulating oxytocinergic neurons in PVN. **(A)** Flowchart illustrating the role of PVN oxytocinergic neurons in regulating cardiac function improvement in mice with myocardial ischemia. **(B)** Effects of PVN oxytocinergic neuron regulation on myocardial injury and fibrosis induced by myocardial ischemia. **(C)** Schematic illustration showing regulation of cardiac function in mice with myocardial ischemia by oxytocinergic neurons in the paraventricular nucleus (PVN) of the hypothalamus. **(D–I)** Effects of regulating oxytocinergic neurons in the PVN on cardiac function indicators in mice with myocardial ischemia. Data are expressed as mean ±standard deviation (*n* = 6 rats/group). * *p* < 0.05 indicates a significant difference between groups.

Chemogenetic inhibition of OT neurons produced therapeutic effects comparable to electroacupuncture, improving myocardial injury, reducing collagen deposition, and enhancing cardiac function. However, activation of OT neurons reversed these beneficial effects of electroacupuncture (Figure 8), suggesting that oxytocinergic neurons in the PVN may serve as a key target in mediating the cardioprotective effects of acupuncture in AMI.

### The downstream projection nuclei of oxytocinergic neurons in PVN as potential future research targets

**Figure 9:**
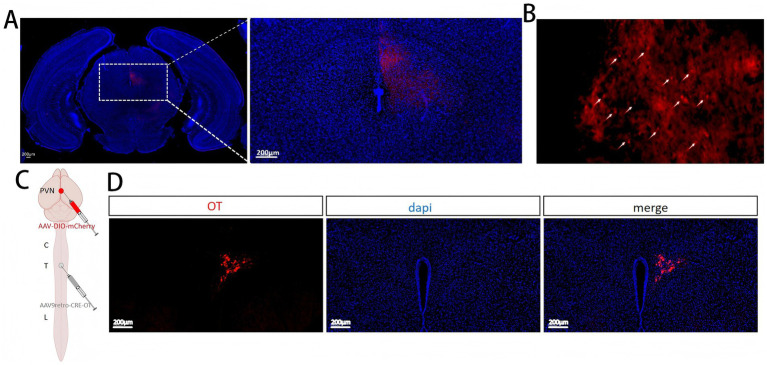
Downstream projection nuclei of PVN oxytocinergic neurons. **(A)** Projection of PVN OT neurons to the PAG. **(B)** Projection of PVN OT neurons to the spinal cord. **(C)** Flowchart of viral tracing experiment verifying PVN projections to the thoracic spinal cord. **(D)** Schematic diagram of the PVN–spinal cord pathway traced from PVN OT neurons.

Brain and spinal cord sections from PVN regions injected with AAV-OT-mCherry virus revealed several downstream fiber projection sites, including the periaqueductal gray (PAG) (Figure 9A). Notably, viral axon expression was observed in the thoracic spinal cord (Figure 9B), a key center for the central regulation of cardiac output. To confirm the existence of this pathway, a viral tracing experiment was conducted. Retro-AAV-cre-OT virus was injected into the T1 thoracic spinal cord, while AAV-hSyn-DIO-mCherry virus was injected into the PVN (Figure 9C), confirming a monosynaptic pathway linking PVN OT neurons to the spinal cord (Figure 9D).

## Discussion

The present study provides compelling evidence for the neuromodulatory role of hypothalamic paraventricular nucleus (PVN) oxytocinergic (OT) neurons in mediating the cardioprotective effects of electroacupuncture (EA) at Shenmen (HT7) in a mouse model of acute myocardial ischemia (AMI).

Our results demonstrate that EA at HT7 significantly improves cardiac function, as indicated by increased ejection fraction (EF) and reduced left ventricular dimensions and volumes, which correlated with histological improvements in myocardial structure. These findings clearly suggest that acupuncture can effectively alleviate myocardial damage caused by ischemia (Kun et al., 2023; Longhurst, 2007). In line with this, a randomized clinical trial conducted by Professor Liang Fengrong’s team, published in JAMA Internal Medicine, confirmed that acupuncture, as an adjunctive therapy to anti-ischemic drugs, significantly reduced the frequency and severity of angina pectoris in 404 patients, with a favorable safety profile. This clinical evidence provides valuable translational insights for the present study (Zhao et al., 2019).

The PVN, as a central hub for autonomic and endocrine regulation, modulates cardiac function via multiple pathways (Schunke et al., 2023; Das et al., 2022; Zheng et al., 2022). It integrates inputs from forebrain regions such as the prefrontal cortex and amygdala (Ge et al., 2024; Obray et al., 2025), and projects fibers to cardiovascular centers in the brainstem, including the rostral ventrolateral medulla (Xu et al., 2025), nucleus tractus solitarius, and dorsal motor nucleus of the vagus. Additionally, the PVN directly influences preganglionic sympathetic neurons, thereby regulating heart rate and myocardial contractility. In this study, inhibition of OT neurons in the PVN reduced heart rate variability (LF/HF), indicating a role in autonomic balance. Previous research has similarly reported that OT neurons can enhance sympathetic activity (Du et al., 2015). OT neurons in the spinal cardiovascular centers have been shown to elevate blood pressure and heart rate (Maier et al., 1998). Moreover, administration of oxytocin into the stellate or middle cervical ganglia increased heart rate and myocardial contractility, suggesting activation of thoracic sympathetic ganglia involved in cardiac regulation (Armour and Klassen, 1990). In the present study, these cardioprotective effects were associated with decreased PVN neuronal firing and specific inhibition of PVN^OT^ neurons. Collectively, these findings establish a functional link between EA-induced neuromodulation and cardiac improvement. Chemogenetic experiments further confirmed a causal neurocardiac axis in which PVN^OT^ neurons are both necessary and sufficient for EA at HT7 to attenuate myocardial ischemia.

The PVN functions as a critical integrator of neural and hormonal signals to maintain cardiovascular homeostasis. Our findings highlight the pivotal role of PVN^OT^ neurons in this process. Under physiological conditions, chemogenetic regulation of PVN^OT^ neurons altered PVN electrical activity and cardiac function, confirming their involvement in baseline cardiovascular regulation. During AMI, activation of PVN^OT^ neurons abolished the cardioprotective effects of EA, emphasizing their role as a therapeutic target. These results provide mechanistic insights into the neurobiological basis of EA’s benefits and suggest that modulation of PVN^OT^ neurons may represent a promising therapeutic strategy for ischemic heart disease.

Investigation of the downstream projections of PVN^OT^ neurons revealed additional mechanisms underlying their effects. Fiber projections were identified in the periaqueductal gray (PAG), which participates in pain and cardiovascular regulation. More notably, viral axons from PVN OT neurons were traced to the thoracic spinal cord, a key center for cardiac output regulation. These observations are consistent with previous findings suggesting that PVN^OT^ spinal projection neurons directly influence cardiac function and may drive sympathetic overactivation after AMI (Roy et al., 2018; Yang et al., 2009). The demonstration of a monosynaptic pathway from PVN^OT^ neurons to the spinal cord further supports this mechanism and strengthens the link to EA-mediated cardioprotection.

The clinical implications of these findings are significant. Acupuncture, as an adjunctive therapy for ischemic heart disease, may provide a non-pharmacological approach to improving cardiac function and reducing the risk of adverse cardiovascular outcomes. By targeting PVN^OT^ neurons, acupuncture could activate cardioprotective pathways and facilitate myocardial repair. Further translational studies are warranted to explore these mechanisms and assess their potential for patient care.

Despite the strengths of this study, several limitations should be acknowledged. These include the relatively small sample size (*n* = 6), use of only male mice, and potential nonspecific effects of AAV-based viral vectors. Additionally, this work was conducted in a murine AMI model, and its direct applicability to humans remains uncertain. Although downstream projections of PVN^OT^ neurons were identified, the precise molecular mechanisms underlying their cardioprotective effects require further clarification. Future research should aim to elucidate the signaling pathways involved and evaluate the therapeutic potential of targeting PVN^OT^ neurons in clinical populations with ischemic heart disease.

In conclusion, this study establishes a novel neurocardiac axis (HT7–PVN^OT^–heart) and provides mechanistic insights into the cardioprotective effects of EA at HT7. By targeting PVN^OT^ neurons, acupuncture emerges as a promising therapeutic approach for ischemic heart disease. Further investigations are required to advance the translational potential of these findings and develop targeted neurocardiac interventions for patients with this condition.

## Data Availability

The raw data supporting the conclusions of this article will be made available by the authors, without undue reservation.
